# Combined 5-FU and ChoKα Inhibitors as a New Alternative Therapy of Colorectal Cancer: Evidence in Human Tumor-Derived Cell Lines and Mouse Xenografts

**DOI:** 10.1371/journal.pone.0064961

**Published:** 2013-06-10

**Authors:** Ana de la Cueva, Ana Ramírez de Molina, Néstor Álvarez-Ayerza, Ma Angeles Ramos, Arancha Cebrián, Teresa Gómez del Pulgar, Juan Carlos Lacal

**Affiliations:** 1 Traslational Oncology Unit, Instituto de Investigaciones Biomédicas, CSIC, Madrid, Spain; 2 Instituto de Investigación Sanitaria IdiPAZ, Madrid, Spain; Enzo Life Sciences, Inc., United States of America

## Abstract

**Background:**

Colorectal cancer (CRC) is the third major cause of cancer related deaths in the world. 5-fluorouracil (5-FU) is widely used for the treatment of colorectal cancer but as a single-agent renders low response rates. Choline kinase alpha (ChoKα), an enzyme that plays a role in cell proliferation and transformation, has been reported overexpressed in many different tumors, including colorectal tumors. ChoKα inhibitors have recently entered clinical trials as a novel antitumor strategy.

**Methodology/Principal Findings:**

ChoKα specific inhibitors, MN58b and TCD-717, have demonstrated a potent antitumoral activity both *in vitro* and *in vivo* against several tumor-derived cell line xenografts including CRC-derived cell lines. The effect of ChoKα inhibitors in combination with 5-FU as a new alternative for the treatment of colon tumors has been investigated both *in vitro* in CRC-tumour derived cell lines, and *in vivo* in mouse xenografts models. The effects on thymidilate synthase (TS) and thymidine kinase (TK1) levels, two enzymes known to play an essential role in the mechanism of action of 5-FU, were analyzed by western blotting and quantitative PCR analysis. The combination of 5-FU with ChoKα inhibitors resulted in a synergistic effect *in vitro* in three different human colon cancer cell lines, and *in vivo* against human colon xenografts in nude mice. ChoKα inhibitors modulate the expression levels of TS and TK1 through inhibition of E2F production, providing a rational for its mechanism of action.

**Conclusion/Significance:**

Our data suggest that both drugs in combination display a synergistic antitumoral effect due to ChoKα inhibitors-driven modulation of the metabolization of 5-FU. The clinical relevance of these findings is strongly supported since TCD-717 has recently entered Phase I clinical trials against solid tumors.

## Introduction

Colorectal cancer (CRC) is the first most prevalent cancer and is the second cause of cancer death in Europe with about 212.000 deaths every year [1]. The most studied drug in CRC is the antimetabolite 5-fluorouracil (5-FU), developed over 50 years ago [2]. 5-FU is an analog of uracil with a fluorine atom. Its mechanism of cytotoxicity consists in misincorporation of fluoronucleotides into RNA and DNA but the main toxic effects are mediated by the inhibition of the nucleotide synthetic enzyme thymidylate synthase (TS). 5-FU is widely used in the treatment of a range of cancers, including CRC, breast and head and neck cancers [3], [4]. Response rates for 5-FU based chemotherapy as a first-line treatment for advanced CRC cancer are only 10–15% [5]. Combination of 5-FU with new cytotoxic drugs such as oxaliplatin and irinotecan has improved the response rates to 40–50% [6], [7]. Furthermore, novel biological agents such as the monoclonal antibodies cetuximab and bevacizumab have demonstrated additional benefits in patients with metastatic disease [8], [9]. Thus, this approach is achieving important improvements, and promotes new therapeutic strategies based on combinatorial treatments.

Choline kinase alpha (ChoKα), the first enzyme in the Kennedy pathway, is responsible for the synthesis of the major phospholipid of the plasma membranes, phosphatidylcholine (PC). Several studies have demonstrated that ChoKα plays an important role in cell transformation and induces *in vivo* tumorogenesis [10], [11]. Furthermore, ChoKα is overexpressed in colon, breast, lung, prostate, ovary and hematological tumors [11]–[16]. Based on these observations, ChoKα has been used as a novel molecular target to develop a new antitumoral strategy. ChoKα inhibitors (ChoKIs) are derivates of the Hemicolinium-3 (HC3) structure, a known choline kinase inhibitor with a high neurotoxicity *in vivo* [17]–[19]. MN58b [20], [21] was identified as a first generation HC3 derivate with potent antiproliferative activity *in vitro* and efficient antitumoral activity *in vivo* in nude mice systems including colon xenografts [10], [21]. MN58b has been used as a model for a new generation of compounds, and a lead molecule to study the mechanism of action of this novel class of antitumor drugs.

A second generation of ChoKα inhibitors has been synthesized to improve the tolerability of ChoKα inhibitors in mice. TCD-717 has been selected among several molecules because it provided the best results *in vitro* and *in vivo* (unpublished results). ChoKα inhibitors are highly specific drugs for tumor cells, since primary cells are reversibly arrested in G1 and are able to recover their growth kinetics once the drug is removed. However, tumor cells are triggered to cell death concomitant to an increase in the intracellular levels of ceramides [22], [23]. Both drugs, MN58b and TCD-717, are derived from Hemicolinium-3, and as such they are both considered competitive inhibitors with choline at the choline binding pocket [24]–[26].

It has been described that the combined use of a choline kinase-specific siRNA and 5-FU, results in a synergistic effect on the reduction of cell proliferation of breast cancer cells [27]. The aim of the present study was to investigate the antitumor efficacy of the combined administration of chemical ChoKα inhibitors and 5-FU, searching for an alternative treatment that would allow to improve 5-FU rate response in CRC treatment and reduce its associated toxicity. The clinical relevance of this new treatment is strongly supported since TCD-717 has been recently approved to enter clinical trials against solid tumours (http://clinicaltrials.gov/ct2/show/NCT01215864).

## Results

### ChoKα levels in human derived colorectal cancer cell lines

**Figure 1:**
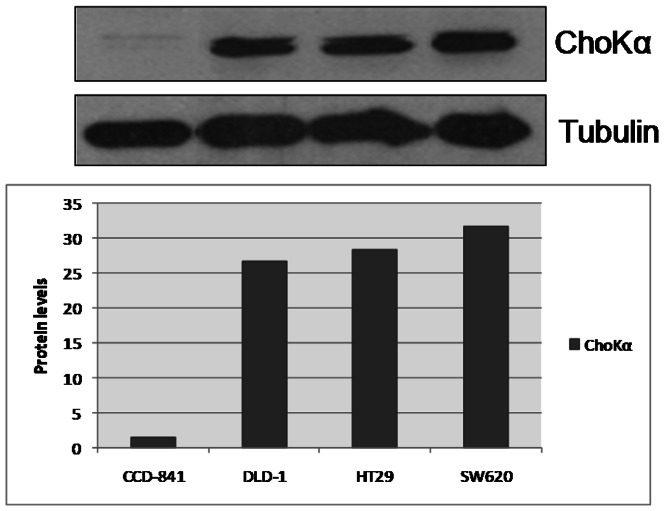
ChoKα expression levels in DLD-1, HT29 and SW620 cell lines by western blot. ChoKα protein levels of three colorectal tumor cell lines, DLD-1, HT29 and SW620 have been compared respect to the non tumoral colorectal cell line CCD-841. Below the western it is represented quantification levels (ChoKα/tubulin).

ChoKα levels were analyzed in the three colon cancer cell lines used in this study, DLD-1, HT29 and SW620 versus a non tumoral colorectal cell line CCD-841. Figure 1 shows that ChoKα levels are about 20–30 times higher than the primary cell line. This result is in keeping with previous analysis of ChoKα expression in tumor samples compared with matched normal tissues from the same patient [11], and provides a rational for the potential use of ChoKα inhibitors in the clinic in combination with standard chemotherapy.

### ChoKα inhibitors synergizes with 5-FU promoting cell death of colon cancer cells

**Figure 2:**
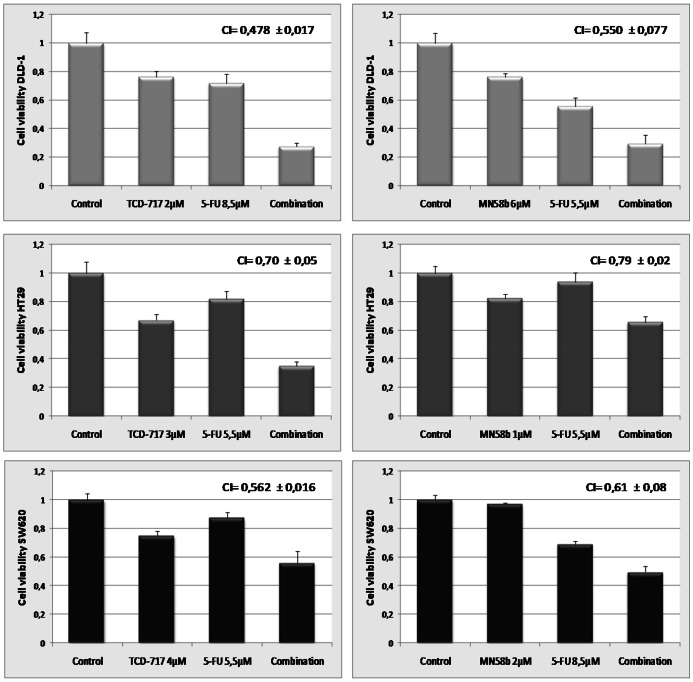
Effect on cell viability of ChoKα inhibitors and 5-FU in DLD-1, HT29 and SW620 cell lines. 6×10^3^ tumor cells were cultured in 96 well plates. After 24 h incubation, cells were exposed to TCD-717(left panels) for 24 h or MN58b (right panels) for 9 h. Thereafter the medium was changed for medium containing 5-FU for 60 h in plates previously treated with MN58b and for 24 h in plates treated with TCD-717. Cell viability was evaluated by MTT assay and represented as percentage of control, untreated cells. CI value in each case is the mean of three independent experiments, each performed in quadruplicates. CI<1 indicates a synergistic effect. A representative experiment of three independent experiments is shown.

The effect on proliferation of ChoKα inhibitors in combination with 5-FU was determined in the three colorectal cancer cell lines: DLD-1, HT29 and SW620. To estimate the appropriate concentrations for each compound, cells were treated with a wide range of concentrations based on their respective IC_50_, alone or in combination. Concentrations used were from 1 to 6 µM (MN58b and TCD717) and 2 to 8.5 µM 5FU both as concomitant and sequential treatments (Fig S1). The best combination to achieve an efficient synergism as antiproliferative drugs was a sequential treatment initiated by a ChoKα inhibitor and followed by 5-FU. The inhibitory effect was quantified by the MTT assay, and the inhibition rates were analyzed by the method of Chou and Talalay and combination indexes (CIs) estimated according to the Calcusyn program [28]. Plots were obtained when the ChoKα inhibitors TCD-717 and MN58b were combined with 5-FU in DLD-1, HT29 and SW620 cell lines (Figure S1). CIs and a representative figure of each different CRC cell line by the sequential combination of ChoKα inhibitors and 5-FU are shown (Figure 2).

**Figure 3:**
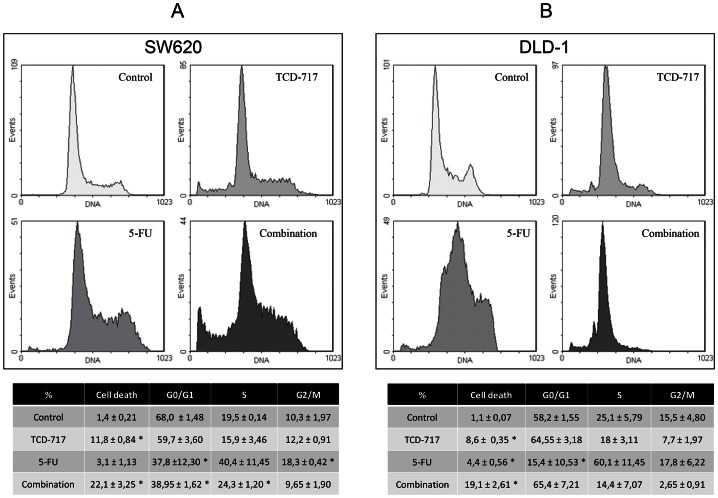
Cell cycle distribution in DLD-1 and SW620 from TCD-717 and 5-FU treatment, alone or in combination. 2,5×10^5^ DLD-1 and SW620 cell lines were seeded in 6 well plates and incubated for 24 h with TCD-717 followed by 5-FU for 24 h alone or in combination. Combination of the two drugs increased cell death compared to the two drugs alone. Tables under the graphics indicate the percentage of the different phases of cell cycle when we treat with TCD-717 and 5-FU alone or in combination. *p<0.05 compared the different cell cycle phases vs. control.

The effect of this combination was investigated, and cell cycle distribution induced by TCD-717 and 5-FU alone or in combination analyzed (Figure 3). Flow cytometry analysis showed a significant induction of cell death after treatment with ChoKIs with a further increase in combination with 5-FU, indicating that the combined treatment had a stronger effect than individual treatments. Previous studies demonstrated that tumour cells are sensitive to ChoKα inhibitors if exposed at G1 phase, but become insensitive in S phase [23]. 5-FU exposure induced S phase accumulation, but the combined treatment drastically reduced S phase accumulation and increased cell death rates. These results support the requirement of a sequential treatment initiated with ChoKα inhibitors explaining the lack of synergism observed with alternative schedules of treatment.

### *In vivo* synergism of ChoKα inhibitors and 5-FU in nude mice

**Figure 4:**
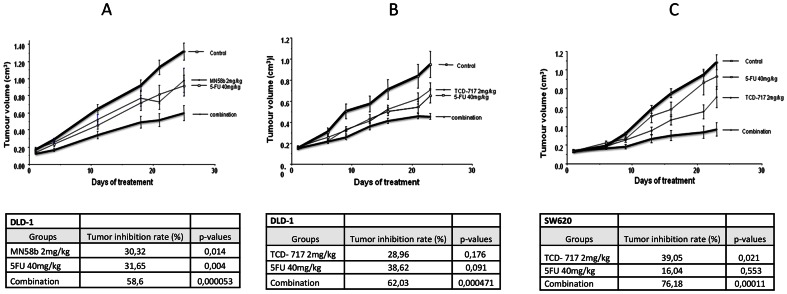
Tumour growth inhibition by combination of ChoKα inhibitors and 5-FU on DLD-1 and SW620 xenografts in athymic nude mice. Mice were exposed to 2 mg/kg/day of ChoKα inhibitors three days a week and 40 mg/kg/day of 5-FU two days a week in combination or alone during 3 weeks. Tumor growth inhibition rate is shown below each experiment. (A) DLD-1 tumors treated with MN58b as ChoKα inhibitor and 5-FU. (B) DLD-1 tumors treated with TCD-717. (C) SW620 tumors exposed to TCD-717 and 5-FU.

The effects of combinatorial treatments of ChoKα inhibitors and 5-FU on the *in vivo* tumor growth of DLD-1 and SW620 xenografts in nude mice were next investigated. DLD-1 xenografts were inoculated into athymic mice and when tumours reached the standard volume of around 0.2 cm^3^, mice were randomly divided into four groups (10 tumors/group) and treated following the next schedule: ChoKα inhibitors were administered at 2 mg/kg/day three times a week during 3 weeks, and 5-FU was administered at 40 mg/kg/day twice a week for 3 weeks. Tumour growth was recorded after the initiation of treatment. Tumour volumes were reduced in all treated groups regardless of the treatment, compared with those of control, untreated mice (Figure 4). Tumor growth of the combination groups in each experiment was significantly smaller than those treated with ChoKα inhibitors or 5-FU alone (p-values<0.05) indicating a strong reduction of tumor volume in the combination schedules.

As a validation, SW620 xenografts were also investigated following an identical schedule with a combination of TCD-717 and 5-FU. A statistically significant effect was also observed in the combination treatment (Figure 4).

### ChoKα inhibitors modulate the expression levels of key enzymes involved in the metabolism of 5-FU

**Figure 5:**
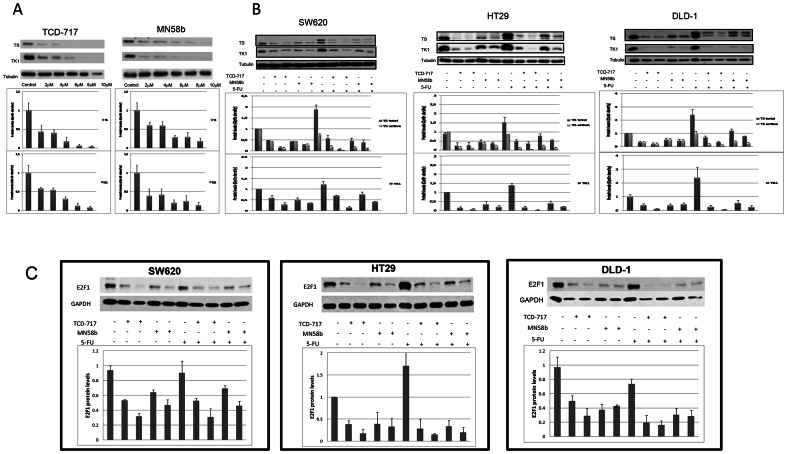
Levels of TS and TK1 in SW620, HT29 and DLD-1 cell lines determined by Western blot analysis. 2,5×10^5^ DLD-1, HT29 and SW620 cell lines were seeded in 6 well plates and incubated for 24 h. (A) SW620 cell line was exposed to increased concentrations of ChoKα inhibitors for 24 h. (B) SW620, HT29 and DLD-1 cell lines were exposed to two concentrations of ChoKα inhibitors [TCD-717: 6 µM (left line) or 10 µM (right line); MN58b: 10 µM (left line) or 15 µM (right line)]. Where indicated, medium was removed after treatment with ChoKα inhibitors as above and changed for fresh medium with 5.5 µM 5-FU for an additional 24 h. Protein expression was analyzed by western blot using monoclonal anti-thymidilate Synthase TS106 clone for TS and monoclonal anti-thymidine kinase clone F12 for TK1. The figure shows on the top a representative blot, at the bottom protein levels (total TS, active TS, and TK1/tubulin) calculated by the mean ± SEM of three independent experiments. (C) 2,5×10^5^ SW620, HT29 and DLD-1 cell lines were seeded in 6 well plates and incubated for 24 h. (A) SW620, (B) HT29 and (C) DLD-1 cell lines were exposed to two concentrations of ChoKα inhibitors [TCD-717: 6 µM (left line) or 10 µM (right line); MN58b: 10 µM (left line) or 15 µM (right line)]. Where indicated, medium was removed after treatment with ChoKα inhibitors as above and changed for fresh medium with 5.5 µM 5-FU for an additional 24 h. Protein expression was analyzed by western blot using anti-E2F1. The figure shows on the top a representative blot, at the bottom protein levels (E2F1/GAPDH) calculated by the mean ± SEM of three independent experiments.

To elucidate the mechanism of this synergistic effect of ChoKα inhibitors and 5-FU, we examined the effect of ChoKα inhibitors on the expression levels of key enzymes in the metabolic pathway of 5-FU such as thymidylate synthase (TS) and thymidine kinase (TK1). SW620 cells were treated with increasing concentrations of ChoKα inhibitors from 2 to 10 µM (Figure 5A) showing a dosage-dependent decrease in the levels of these proteins. Next, SW620, HT29 and DLD-1 were treated with ChoKα inhibitors (TCD-717 6 and 10 µM, MN58b 10 and 15 µM) and 5-FU (5.5 µM), alone or in sequential combination to determine the effects on the expression levels of these enzymes (Figure 5B). Free (active form) and ternary complex (inactive form) of TS and TK1 were analyzed by western blotting. As expected, cells treated with 5-FU showed both the TS ternary complex (upper band) and the free TS (lower band). ChoKα inhibitors induced a significant down-regulation of both free TS (active form) and ternary complex (inactive form), as well as TK1 (Figure 5B). Thus the sequential combination of ChoKα inhibitors and 5-FU induced both mechanisms of inactivation, decreased formation of ternary complex and a significant down-regulation of TS active (free form). In addition, ChoKIs decreased TK1 levels, affecting also the mechanism associated to 5-FU resistance. This effect could explain the mechanism of synergism between both drugs.

Both TK1 and TS have been shown to be under transcriptional control of E2F1 [29], [30]. Thus we investigated whether the drastic reduction in the levels of TK1 an TS after ChoK inhibition was mediated by an effect on the levels of this transcription factor. As shown in Figure 5C, in all three cell lines investigated, a drastic reduction in the levels of E2F1 was observed consistent with its role as regulator of TS and TK1 synthesis.

**Figure 6:**
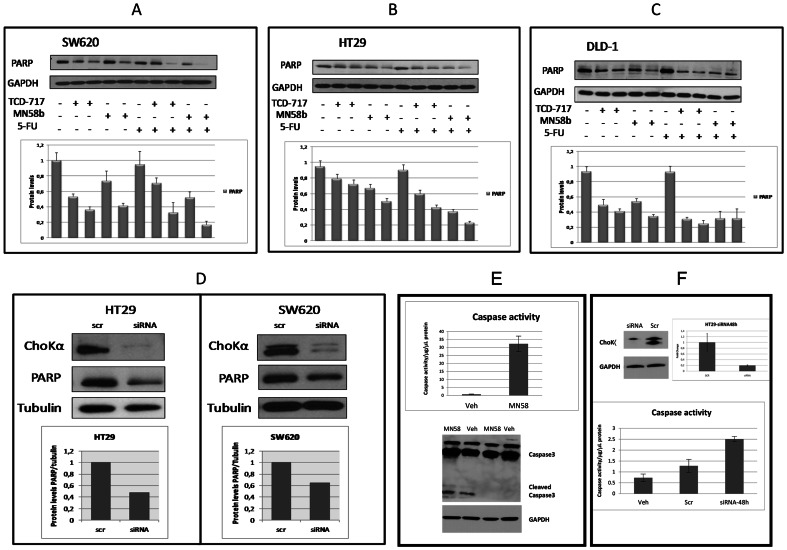
Induction of apoptosis in SW620, HT29 and DLD-1 cell lines after treatment with ChoKIs. (A–C) SW620, HT29 and DLD-1 cells were seeded as described under Material and Methods and treated with two concentrations of ChoKα inhibitors [TCD-717: 6 µM (left line) or 10 µM (right line); MN58b: 10 µM (left line) or 15 µM (right line)] for 24 h and, where indicated, with 5.5 µM 5-FU or vehicle for an additional 24 h. Protein expression was analyzed by Western blot using polyclonal antibody anti-PARP. Behind each graph, data shows protein levels (PARP/GAPDH) calculated by the mean ± SEM of three independent experiments. (A) SW620. (B) HT29. (C) DLD-1. (D) PARP and ChoKα expression levels in HT29 and SW620 cell lines transfected with a specific ChoKα siRNA determined by Western blot analysis or a control scramble siRNA (scr). Protein expression was analyzed by Western blot using ChoKα monoclonal antibody and polyclonal antibody anti-PARP. Below each Western the ratio PARP/Tubulin is represented. (E) Caspase 3 activity (upper panel) and its cleavage (lower panel) were determined in HT29 cells after treatment with MN58b for 24 h. (F) Caspase 3 enzymatic activity was also measured after transfection of HT29 cells with a ChoKα specific siRNA or a control siRNA (scr) for 48 h. Western blot represents the levels of ChoKα after transfection and its relative reduction normalized to GAPDH levels.

Next, to investigate the potential induction of apoptosis after ChoK inibition, as a hallmark of apoptosis, the levels of PARP were analyzed under similar conditions, and a drastic reduction was observed in PARP levels in SW620 (Figure 6A), HT29 (Figure 6B) and DLD-1 (Figure 6C) cell lines after treatment with the combination schedule. PARP is a protein implicated in DNA repair and its decrease has a similar physiological meaning to that of its cleavage because reduced levels of PARP avoid DNA repair and cells are triggered to apoptosis engagement.

HT29 and SW620 cells were also transfected with a specific siRNA-ChoKα. As shown in Figure 6D, similar results were obtained to those of ChoKα pharmacological inhibition, with a drastic reduction in PARP levels, an indication of specificity due to ChoKα inhibition.

As an additional evidence of the induction of apoptosis after ChoK inhibitors in colon cancer cells, two alternative methods for the initiation of the apoptotic signaling machinery were used. Caspase 3 activity and cleavage was readily detected after MN58b treatment of HT29 cells (Figure 6E). Downregulation of ChoKα expression by specific siRNA also increased caspase activity, a further support of the specificity of this effect based on reduction of ChoKα activity (Figure 6F). Thus, ChoK inhibitors are able to induce apoptosis by the activation of caspase 3.

**Figure 7:**
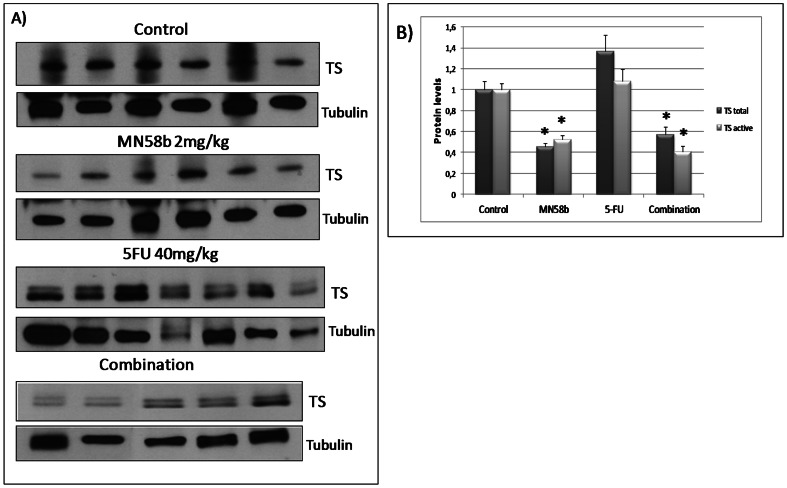
TS expression levels in tumor tissues from DLD-1 xenografts after treatment with MN58b plus 5-FU. Mice were inoculated with DLD-1 cells as indicated under Materials and Methods and either left untreated, or treated with MN58b or 5-FU alone or in combination. A) TS expression levels of each experiment group: Control, MN58b, 5-FU and combination group. Tubulin was used as loading control. B) Graph represents mean protein levels as TS/tubulin ratios for each group. Black bars represent total TS/tubulin values; Grey bars represent TS active values. (*) Statistically significant (p<0.05), compared to control group.

Finally, TS levels were analyzed in tumors generated from the *in vivo* xenograft model DLD-1 after treatment with MN58b and 5-FU and compared to control, untreated mice (Figure 7). A statistically significant reduction in TS levels (both total and active protein) was observed in tumors treated with MN58b alone or in combination with 5-FU, while no significant difference was found in the tumors from mice treated with 5-FU alone.

### Down-regulation of mRNA levels of TS and TK1 gene after treatment with ChoKα inhibitors and 5FU in DLD-1, SW620 and HT29 cells

Preclinical and clinical studies have demonstrated that TS expression is determinant of 5-FU sensitivity and clinical outcome [31], [32]. In keeping with these results, gene amplification of TS with consequent increases in TS mRNA and protein, has been observed in cell lines that are resistant to 5-FU [31], [33], [34].

**Figure 8:**
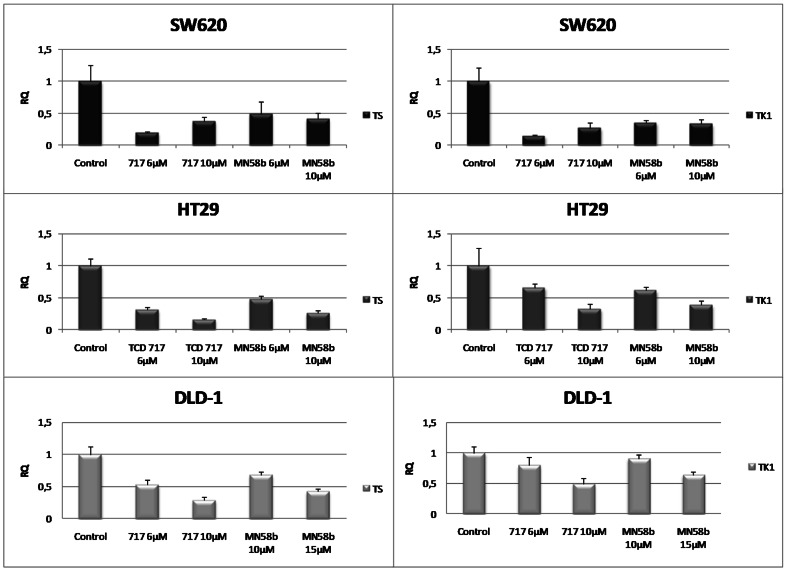
Levels of TS and TK1 in SW620, HT29 and DLD-1 cell lines determined by RT-qPCR. 2,5×10^5^ DLD-1, HT29 and SW620 cell lines were seeded in 6 well plates and incubated under optimal conditions for 24 h. Next, cells were exposed to different concentrations of ChoKα inhibitors for 24 h. Levels of TS and TK1 were analyzed by RT-qPCR as described under Materials and Methods. 18S was used as the endogenous control for normalisation.

Based on the above results, SW620, HT29 and DLD-1 cells were treated with MN58b and TCD-717 for 24 h. Treatment with ChoKα inhibitors resulted in a significant down-regulation of both TS and TK1 mRNA levels as determined by real time Q-PCR assay using the specific probes Hs00426591_m1 for TS and Hs01062125_m1 for TK1 (Figure 8). These results indicate that the down modulation in the protein levels of TS and TK1 induced by ChoKα inhibitors results from a drastic reduction at the transcriptional level, with a significant effect in both TS, and its salvage pathway mediated by TK1. Since the levels of E2F1 were also drastically reduced, it is reasonable to propose that this transcription factor is responsible for the observed effects on TS and TK1 inhibition as previously described [29], [30].

**Figure 9:**
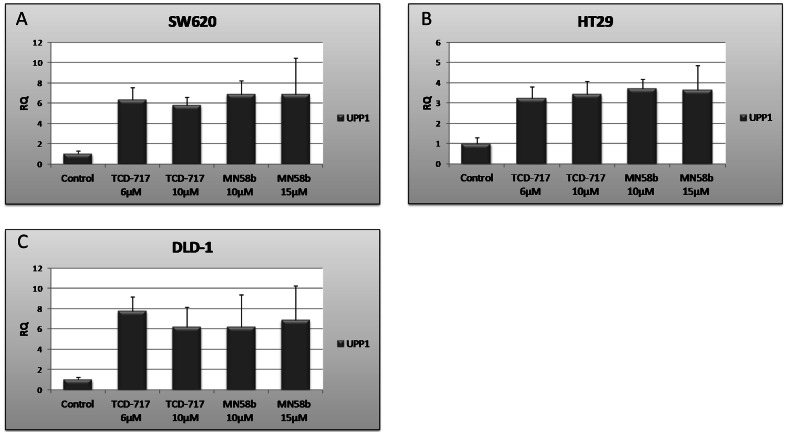
*UPP1* mRNA in SW620, HT29 and DLD-1 cell lines. 2,5×10^5^ DLD-1, HT29 and SW620 cell lines were seeded in 6 well plates and incubated 24 h under standard conditions. Thereafter, cells were treated with ChoKα inhibitors, TCD-717 (6 and 10 µM) and MN58b (10 and 15 µM) during 24 h. 18S was used as an endogenous control.

The above results would be consistent with a general effect on gene transcription induced by ChoKα inhibitors. To test for this possibility, mRNA levels of other proteins also involved in 5-FU mechanism of action were investigated. Contrary to what was observed with TS or TK1, uridine phosphorylase (*UPP1*) was increased after treatment with ChoKα inhibitors (Figure 9). This elevation may be physiologically relevant because *UPP1* is responsible for the transformation of 5-FU to an intermediate metabolite that will be converted into the active metabolites fluorouridine triphosphate (FUTP) and fluorodeoxyuridine triphosphate (FdUTP). Thus, the effects observed on E2F1, TS and TK1 do not respond to a general, non-specific effect on gene transcription.

### Effects of ChoKα inhibitors on MAPK, AKT signaling and ceramides levels

**Figure 10:**
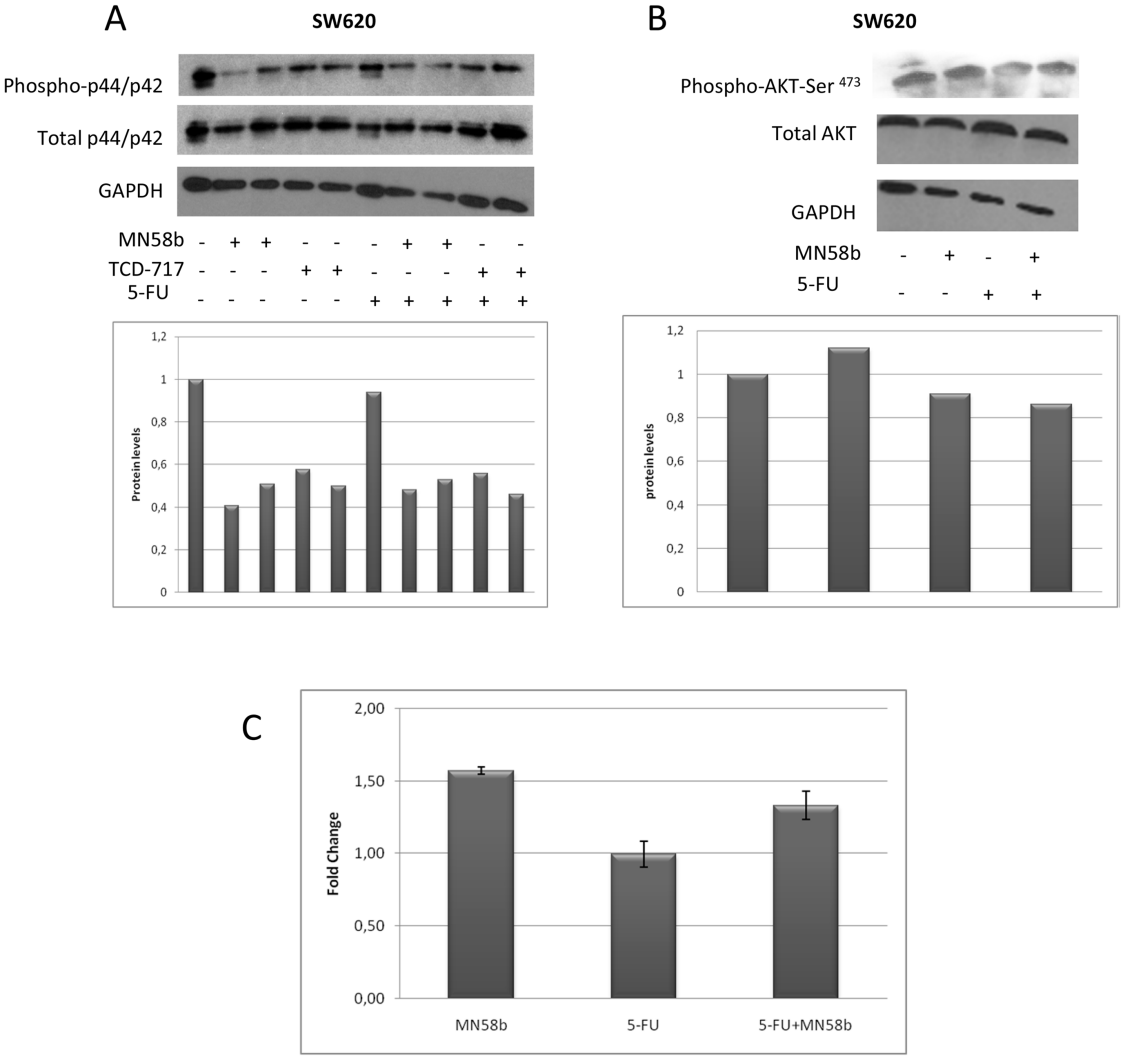
Effects of ChoK inhibition on p44/p42 MAPK levels and ceramide levels. (A) 2,5×10^5^ SW620 cells were seeded in 6 well plates and incubated for 24 h and exposed to two concentrations of ChoKα inhibitors [TCD-717: 6 µM (left line) or 10 µM (right line); MN58b: 10 µM (left line) or 15 µM (right line)]. Where indicated, medium was removed after treatment with ChoKα inhibitors and changed for fresh medium with 5.5 µM 5-FU for an additional 24 h. Protein expression was analyzed by western blot using monoclonal anti-phospho p44/p42 MAPK and anti-p44/p42 MAPK. The figure shows a representative blot of two independent experiments. (B) SW620 cells were grown and treated as indicated in (A) with 15 µM MN58b and 5.5 µM 5-FU alone or in combination for 24 hours. Protein expression was analyzed by western blot using anti-pAKT- Ser^473^, anti-AKT or anti-GAPDH. The figure shows on the top a representative blot, at the bottom protein levels (pAKT-Ser^473^/total AKT/GAPDH) from a representative experiment. (C) 2×10^6^ SW620 cells were seeded in p100 plates and incubated 24 h under standard conditions. Cells were then treated with 15 µM MN58b and 5.5 µM 5-FU alone or in combination for 24 hours. Total ceramide levels were analyzed by UPLC-TOF. Mean ± SD of two independent experiments performed in triplicate are shown.

As previously described by us and Chesney's group [40]–[42] inhibition of ChoKα has an effect on the MAPK kinase and AKT signaling pathways with a drastic reduction in the phosphorylated forms in several cell types. In keeping with these results, when SW620 cells were treated with ChoKα inhibitors, a drastic reduction in both p42/p44 was observed (Figure 10A). However, in contrast with previous reports on other cell lines, no significant effect was observed in the phosphorylation of pAKT-Ser^473^ (Figure 10B). Furthermore, no effect on either signaling pathway was observed by treatment of SW620 cells with 5-FU alone, and no significant modification of the effects observed when cells were treated with ChoKα inhibitors was observed under combination conditions.

Finally, generation of ceramides has been observed specifically in Jurkat cells after treatment with MN58B while no significant increase was observed in human primary lymphocytes [22]. This may be responsible for the induction of apoptosis in this cellular system and could also explain the observed synergism in this study. However, no effect on ceramides levels were observed after 5-FU treatment alone nor after combinatorial schedules with MN58B and 5-FU (Figure 10B), excluding this mechanism as responsible for the synergism observed when 5-FU was combined with ChoK inhibitors.

## Discussion

Colon cancer is one of the most common causes of death from cancer worldwide. The standard treatment for CRC is based on 5-FU as first-line usually in combination with other cytotoxic drugs such as oxaliplatin or the topoisomerase I inhibitor CPT-11 (Irinotecan) [33]–[35].

A better knowledge of the molecular biology of CRC tumors has changed management of CRC patients, with the status of K-Ras mutations being a critical decision-making issue. Numerous clinical trials are in progress to improve schedules for CRC patients, as for many other types of cancer. However, the low rates of curative treatments available makes still necessary to explore new therapeutic approaches to achieve better rates in survival. In this sense, a very active search for novel treatments based on combinatorial chemotherapy and targeted therapy has generated new promising alternatives. This strategy is based on schedules that have raised response rates from 10-15% with 5-FU alone to 40–50% in combination chemotherapy [6], [7].

Targeted therapy is a treatment with a focused mechanism that specifically acts interfering a well-defined target or biological pathway [36]. Currently, these new approaches constitute a real promise for improved management and outcome of cancer patients, since it specifically acts against cancer cells and therefore should have less side effects than other types of therapeutic treatments.

ChoKα is overexpressed in a large variety of human tumors including colorectal cancer [11]–[16]. Increased levels of total choline metabolites, including phosphocholine, the product of ChoK activity, is also a common feature of many types of tumors [37]. Therefore, ChoKα is a novel interesting target for the development of cancer therapies. As a consequence, ChoKα inhibitors have been synthesized to specifically interfere with its activity [10], [18], [20], [21].

The combination of siRNA-ChoK with 5-FU in breast cancer cells has shown a reduction of cell viability and proliferation [27]. However, this treatment relies on the use of a siRNA which is not transported to the target cells *in vivo* in an efficient manner. Here, we use two chemical inhibitors specifically designed against ChoKα (MN58b and TCD-717) with high potency against tumor cells *in vitro* and *in vivo*, to explore the effect of the combination of 5-FU and this new targeted therapeutic approach to improve current CRC treatments. A strong synergistic antitumoral effect when 5-FU and ChoKα inhibitors were used in combination against several CRC cell lines is reported.

Previous reports from our group demonstrate that induction of apoptosis by inhibition of ChoKα is related to the specific generation of ceramides in tumor cells [22], [23]. A dramatic difference in the response to specific ChoKα inhibitors has been observed between normal and tumor cells. Whereas blockage of *de novo* phosphocholine (PCho) synthesis by MN58b in primary cells induces Retinoblastoma (Rb) dephosphorylation and results in reversible cell cycle arrest in G0/G1 phase, tumor cells suffer a drastic wobble in the metabolism of main membrane lipids phosphatidylcholine and sphingomyelin, resulting in a significant increase in the intracellular levels of ceramides that promotes apoptosis [22], [23]. These initial experiments have been reproduced in additional cell types including colon cancer derived cell lines (data not shown). Additional evidence has been reported that indicates a clear inhibition of the PI3K/AKT and ERK signaling pathways by ChoK inhibitors [38], [39], [40]. However, in the cellar systems used in this study, neither MAPK nor AKT signaling or ceramides generation are significantly altered by 5-FU treatment alone. No significant effect is observed either when cells were treated with the combinatorial regimes that showed a potent synergism both under in vitro or in vivo conditions. These results altogether indicate that the previous effects reported on the mechanisms of action of ChoKα inhibitors either on MAPK, AKT and ceramides signaling are not responsible for the synergistic effect observed in colon cancer cells when combining ChoKα inhibitors and 5-FU.

Here we have further investigated the mechanism of the synergism of ChoK inhibitors and 5-FU for a better understanding and improvement of its potential use in the clinic. TS protein has a central role in the biosynthesis of thymidylate, an essential precursor for DNA synthesis [41]. Some studies have shown that TS protein levels are higher in several tumors tissues compared with their normal counterparts [42]. Furthermore, high TS levels are associated with poor prognosis in these cancers [43]–[45]. Another critical enzyme for thymidine metabolism is TK1 which increased activity represents a potential mechanism of resistance to 5-FU [46].

Here, we demonstrate that ChoKα inhibitors induce a strong down modulation of the levels of both TS and TK1, contributing to the induction of apoptosis triggered by 5-FU treatment. Thus, the mechanistic basis for the observed synergism seems to rely on the finding that ChoKα inhibitors are modulating the levels of TS and TK1 enzymes which are related to 5-FU metabolization.

These effects were observed in all three cell lines tested, a strong support to the given explanation on the effects on TS and TK1. Furthermore, IC_50_ values of ChoKIs for all cell lines were found similar in a set of colon cancer derived cells lines regardless of the p53 or K-Ras status. Thus, DLD-1 (wild type p53 and mutated K-Ras, IC_50_ = 2.3 µM), SW620 (mutated p53 and mutated K-Ras, IC_50_ = 3.5 µM), HT29 (mutated p53 and wild type K-Ras, IC_50_ = 1.8 µM), and HCT116 cells (wild type p53 and mutated K-Ras, IC_50_ = 2.5 µM) showed very similar IC_50_ values to TCD-717 treatment.

TS is a key enzyme in the synthesis of DNA and the target enzyme of 5-FU. Several studies demonstrate that the expression levels of TS in the tumoural tissue predicts overall survival for colon cancer and correlates with resistance to 5-FU [47], [48]. Acquired resistance to 5-FU is caused by overproduction of TS resulting from gene amplification. The free form is active and its expression is inversely correlated with the drug sensitivity in several human cancers [48]–[50].

As an attempt to understand the mechanism by which ChoKα inhibitors control the transcriptional levels of TS and TK1, we have found that its transcriptional regulator, E2F1, is also drastically reduced after treatment with ChoKα inhibitors. Although this explains the observed effects on TS and TK1 expression levels, it is an intringuing observation, but it seems that it is not related to a general inhibitory effect on transcription since other enzymes involved in 5-FU metabolization such as UPP1 is not affected in a similar manner. Other enzymes are also not inhibited but induced under similar conditions (data not shown) further supporting a specific role of E2F1 downmodulation in the regulation of TS and TK1 synthesis and the synergism found between ChoKα inhibitors and 5-FU.

The implication of ChoKα inhibitors in the mechanism of action of 5-FU shown here is in keeping with the observed optimal sequential schedule, since treating cells with ChoKα inhibitors first improve the down-regulation of the ternary complex formed by 5-FU. Thus, reducing the enzymes responsible for 5-FU inactivation with ChoKα inhibitors potentiates 5-FU efficacy. ChoKα inhibitors decrease TS protein levels, though the remaining protein is still active. When cells are treated with 5-FU, the remaining active TS form is inactivated, forms the ternary complex avoiding thymidine formation and driving to DNA damage. Thus, with this schedule TS activity is modulated following two different mechanisms, down-regulation of gene expression and inactivation of the protein. However, treating with 5-FU results only in a partial inactivation of the enzyme.

One of the major problems for the success of chemotherapy is drug resistance. Regarding 5-FU resistance, a salvage pathway has been reported in which TK1 plays an important role [48]. We demonstrate that ChoKα inhibitors not only promote 5-FU action by down-regulating the expression of TS, but also block the salvage pathway by down-regulating TK1, preventing dTMP synthesis and its incorporation into DNA.

Our results provide the basis to support a new therapy focused on the combination of ChoKα inhibitors, which are already in Phase I clinical trials, and 5-FU as a new approach for colorectal cancer patients. In addition, this study suggests a promising role for ChoKα inhibitors as a new treatment for patients who had failed 5-FU chemotherapy or display high expression levels of TS or TK1. This strategy may also be used in other types of pathologies where 5-FU constitutes the basis for chemotherapeutic intervention.

## Materials and Methods

### Cell lines and cell proliferation assays

Proliferation studies of MN58b, TCD-717 and 5-FU were determinate using DLD-1, HT29, SW620 and HCT116 CRC cell lines and the non-tumourigenic CCD-841 cell line, all were purchased from the ATCC (Manassas VA, USA). HT29 and SW620 were maintained in DMEM, CCD-841 in MEM, HCT116 in McCoy's medium, and DLD-1 in RPMI1640, supplemented with 10% fetal bovine serum. 6000 cells/well were seeded into 96- well flat-bottom plates (BD, Falcon, Bioscience, San Jose, CA, USA) and incubated for 24 h under standard conditions. P53 and K-Ras status were considered as previously described [51]–[53]. MN58b or TCD-717 were added at different concentrations from stocks solutions. After treatment with ChoKα inhibitors, medium was removed and replaced with fresh medium containing 5-FU. Quantification of the number of cells remaining in each well was carried out by the MTT (3-(4,5-dimethylthiazol-2-yl)-2,5-diphenyltetrazolium bromide) method. Absorbance is read at 560 nm in a VersaMax Microplate Reader (Molecular Devices, Sunnyvale, CA, USA).

### Chemicals

MN58b was dissolved in sterilized H2O; TCD-717 was dissolved in DMSO: H_2_O (v/v, 2∶1), the source of both drugs is Medicinal Chemistry Department, University of Granada, Spain. 5-FU was purchased from Sigma Chemical Co. and reconstituted in PBS. Stock solutions were prepared at 5 mM.

### Combined effect evaluation

Drug interaction between ChoKα inhibitors and 5-FU was assessed using the combination index (CI) [28] where CI<1, CI = 1 and CI>1 indicate synergistic, additive or antagonistic effects respectively. The CI value was calculated according to the formula CI  =  (D1/(D_f_)_1_+ D2/(D_f_)_2_)_,_ where (D_f_)_1_ is the concentration of MN58b or TCD-717 and (D_f_)_2_ the concentration of 5-FU respectively, required to inhibit cell growth x% and D_1_ and D_2_ are the drugs concentrations in combination treatment that also inhibit cell growth by x%. Data analysis was performed by the Calcusyn software (Biosoft, Oxford, UK).

### Flow cytometric assay

Cell cycle distribution was determined by DNA content analysis after propidium iodide (PI) staining. Cells were treated with ChoKα inhibitors and 5-FU alone or in combination for 48 h. Cells were then harvested, stained and incubated with detergent and 1 ml of PI (50 µg/ml). The DNA content of approximately 4×10^5^ stained cells was analyzed using a Coulter XL-MZL flow cytometer. The fraction of cell death, G0-G1, S and G2-M phases were analyzed by DNA program software Multicycle AV for Windows de Phoenix Flow Systems.

### Tumor xenograft studies

All experiments concerning living laboratory animals were performed after protocol approval by a local ethical committee, following Spanish Laboratory Animal. Female athymic BALB/C nude mice were supplied by Jackson Laboratories (Bar Harbor, Maine 04609 USA). MN58b and 5-FU were dissolved in PBS and injected i.p. in amounts of 0.1 ml/mouse. TCD-717 was dissolved in DMSO: H_2_O 2∶1, and diluted with PBS to the appropriate concentration.

Mice were inoculated subcutaneously with injections of 1×10^6^ DLD-1 cells in each flank of the mouse mixed with matrigel (354234, BD Bioscience) 1∶1. Tumor sizes were determinate using micrometer calipers and when the size of the tumors was approximately 0.2 cm^3^, mice were divided into four groups: control group; MN58b 2 mg/kg/3 days group; 5-FU 40 mg/kg/2 days group and MN58b plus 5-FU combination group during 3 weeks. The experiment was repeated following a similar protocol with TCD-717 2 mg/kg/3 days in DLD-1 and SW620.

### Western blot analysis

Cells were incubated with ChoKα inhibitors and 5-FU single or in combination. Same amounts of protein (30 µg) were loading into SDS-PAGE acrylamide gels and resolved proteins were transferred onto nitrocellulose membranes. The following antibodies were used: monoclonal anti thymidylate synthase (TS) TS106 clone (MAB 4130 Millipore), monoclonal anti thymidine kinase (TK1) F12 clone (Sigma, SAB1406531), polyclonal anti PARP (Santa Cruz Biotechnology, sc-7150), polyclonal anti caspase-3 (Cell Signaling, #9662), monoclonal anti ChoKα, polyclonal anti-E2F1, clones KH20 y KH95 (Millipore, 05-379), monoclonal anti-phospho p44/p42 MAPK (Cell Signaling #9106), monoclonal anti p44/p42 MAPK (Cell Signaling #4695), monoclonal anti pAKT (Ser^473^) (Cell Signaling #4051s), and monoclonal anti AKT (Cell Signaling #2920s). Monoclonal anti GAPDH (Millipore, MAB374) and monoclonal anti α-tubulin (Sigma T9026) were used as loading controls.

### siRNA Transfection

4×10^5^ HT29 and SW620 cell lines were transfected using Lipofectamine™ 2000 (Invitrogen) following the manufacturer's instructions. siRNA targeting ChoKα was purchased from Qiagen (SI03063942) and non-targeting control (scr) was purchased from Ambion (AM4635). HT29 and SW620 cells were seeded in 6 wells plates. 24 h later cells were transfected with a ChoKα specific siRNA or a control siRNA (scr) during 24 h in medium OPTI-MEM and then, medium was removed and changed for medium with serum. Proteins were extracted 48 h later. Protein expression was analyzed by Western blot using ChoKα monoclonal antibody, polyclonal antibody anti-PARP or polyclonal antibody anti caspase-3. Monoclonal antibody anti α-tubulin or monoclonal antibody anti GAPDH were used as loading controls.

### Quantitative real-time reverse transcription-PCR analysis

Cells were incubated for 24 h with ChoKα inhibitors. Total RNA was extracted using RNeasy Mini kit (Qiagen). 1 µg of RNA was used to generate cDNA using High-Capacity cDNA Archive Kit (Applied Biosystems), and quantitative real-time PCR was carried out in triplicate using the ABI PRISM 7700 Sequence Detector (Applied Biosystems). 18S ribosomal RNA was amplified as internal control. Probes used for amplification were from Applied Biosystems as Taqman Gene Expression Assays Hs00426591_m1 for *TS*, Hs01062125_m1 for *TK1*, Hs00427695_m1 for *UPP1* and 14319413E for 18S.

### Caspase activity assay

Caspase-3 enzymatic activity was determined using Caspase-3 Fluorometric Assay Kit (R&D Systems, Inc. Minneapolis MN), according to manufacturer's protocol. HT29 cells were seeded in 60 mm dishes at 5×10^5^ cells/dish and treated with MN58b or vehicle for 24 h and then lysed for the assay. Alternatively, cells were transfected with siRNA targeting ChoKα or a control (scr) siRNA as described above and then lysed for the assay.

### Ceramide analysis by UPLC-MS

SW620 cells were seeded at a density of 2×10^6^ cells per p100 plate. Twenty-four hours later, cells were treated with MN58b and 5-FU alone or in combination for 24 hours. Then, cells were washed in PBS and collected by brief trypsinization. Sphingolipid extracts, fortified with internal standards (N-dodecanoylsphingosine, N-dodecanoylglucosylsphingosine, D-erythro-dihydrosphingosine, N-dodecanoylsphingosylphosphorylcholine, and C17D-erythro-dihydrosphingosine-1-phosphate, 0.2 nmol each), were prepared and analyzed by ultraperformance liquid chromatography, coupled to high-resolution electrospray ionization time-of-flight mass spectrometry (UPLC-TOF; Waters, Milford, MA, USA).

## Supporting Information

Figure S1 **Plots obtained by Calcusyn program when ChoKα inhibitors, TCD-717 and MN58b, are combined with 5-FU in the human colorectal cancer cell lines DLD-1, HT29 and SW620.** Graphs represent combination indexes (CI). Numbers represents different experiments exposed in the table below each figure.
(TIFF)
