# Supplementary figures and images for: Combined 5-FU and ChoKα Inhibitors as a New Alternative Therapy of Colorectal Cancer: Evidence in Human Tumor-Derived Cell Lines and Mouse Xenografts
Source: PLoS One. 2013 Jun 10;8(6):e64961. doi: 10.1371/journal.pone.0064961 (PMC3677921; doi:10.1371/journal.pone.0064961)

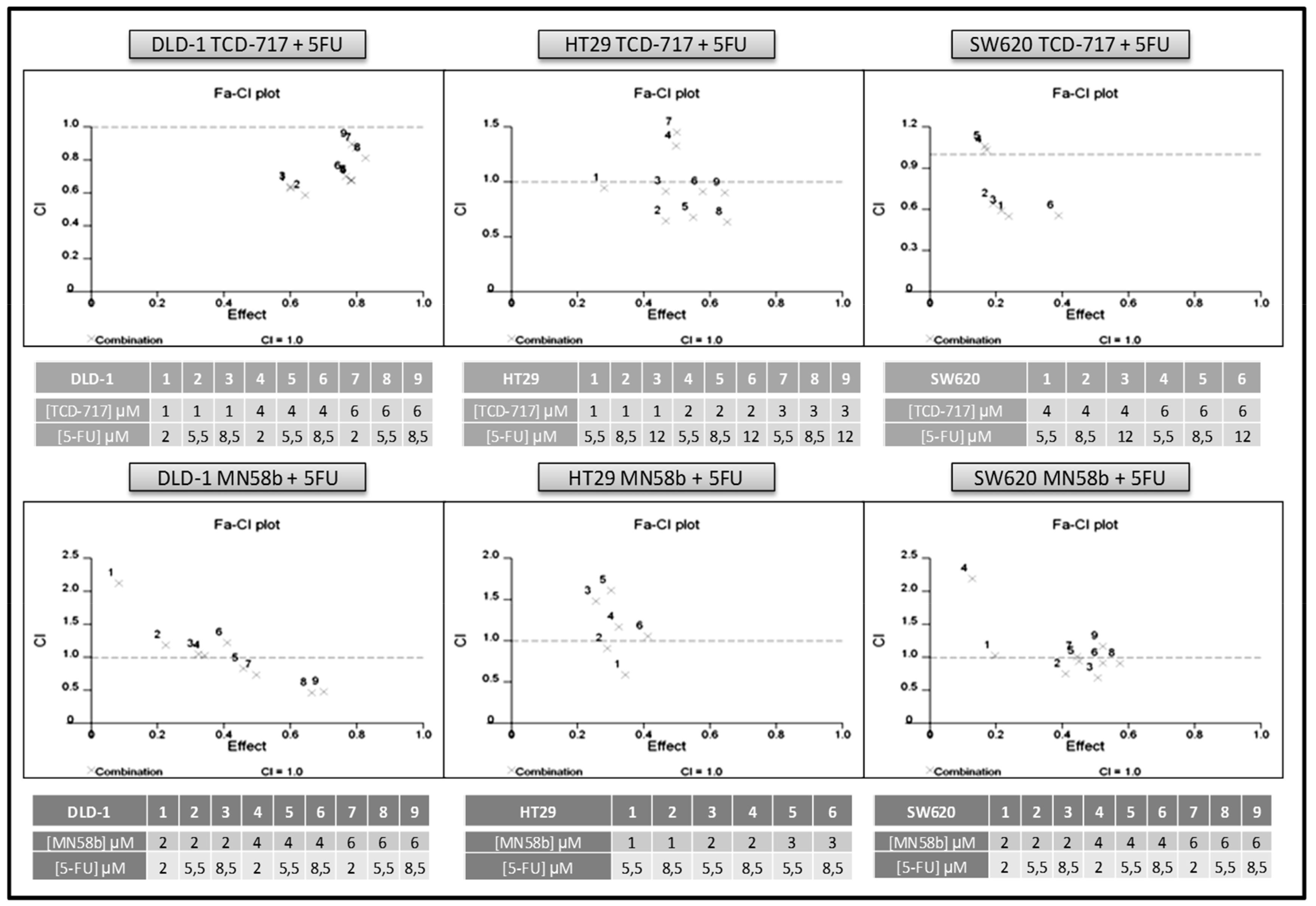

Supplement: Figure S1 — Plots obtained by Calcusyn program when ChoKα inhibitors, TCD-717 and MN58b, are combined with 5-FU in the human colorectal cancer cell lines DLD-1, HT29 and SW620. Graphs represent combination indexes (CI). Numbers represents different experiments exposed in the table below each figure. (TIFF) [file pone.0064961.s001.tif]
